# Towards responsive policy and actions to address non-communicable disease risks amongst adolescents in Indonesia: insights from key stakeholders

**DOI:** 10.1016/j.lansea.2023.100260

**Published:** 2023-08-16

**Authors:** Karly I. Cini, Nisaa R. Wulan, Dorothea Dumuid, Alifah Nurjannah Triputri, Iffat Abbsar, Luo Li, Diandra A. Priambodo, Grace E. Sameve, Artha Camellia, Kate L. Francis, Susan M. Sawyer, George C. Patton, Ansariadi Ansariadi, Peter S. Azzopardi

**Affiliations:** a Global Adolescent Health Group, Maternal Child and Adolescent Health Program, Burnet Institute, Melbourne, Australia; b Centre for Adolescent Health, Murdoch Children's Research Institute, Melbourne, Australia; c Department of Paediatrics, Faculty of Medicine, Dentistry, and Health Sciences, University of Melbourne, Melbourne, Australia; d Alliance for Research in Exercise, Nutrition and Activity (ARENA), Allied Health & Human Performance, University of South Australia, Australia; e Centre for Epidemiology and Population Health Studies, Faculty of Public Health, Hasanuddin University, Makassar, Indonesia; f Faculty of Medicine, Nursing and Health Sciences, Monash University, Melbourne, Australia; g Faculty of Medicine, University of Indonesia, Jakarta, Indonesia; h UNICEF Indonesia, Jakarta, Indonesia; i Adolescent Health and Wellbeing Program, Telethon Kids Institute, Adelaide, South Australia, Australia

**Keywords:** Adolescents, Indonesia, Qualitative, Non-communicable disease, Risk factors

## Abstract

**Background:**

Non-communicable diseases (NCDs) such as cancer, diabetes, heart disease, mental disorder and chronic lung conditions are the leading cause of death and disability in Indonesia. Adolescence is when risks for NCDs emerge and it is also an important life stage for intervention, yet young people are often at the margins of NCD policy and actions. This study aimed to understand how policies and actions should address NCD risks (tobacco smoking, inadequate physical activity, and diet) for adolescents in Indonesia, and how young people can be meaningfully involved.

**Methods:**

Qualitative in-depth interviews over videoconference (n = 21) were conducted in English or Bahasa with stakeholders in Indonesia. Participants included policymakers, implementation partners, and advocates who were focused on adolescent health or NCDs. Interviews were recorded, transcribed, translated, and thematically analysed using NVivo12. Findings were disseminated to participants for validation and feedback. Youth participants (n = 7) attended an additional workshop and considered recommendations and actions arising from this research.

**Findings:**

Participants identified that government and non-government organisations are acting on NCDs in Indonesia, but few of the existing initiatives target adolescents, and adolescent services rarely addressed NCD risks. Participants also felt that policies to protect adolescents from NCD risks (i.e., smoke-free areas in public) were not always enforced. For programs or initiatives focused on adolescent health, those that had engaged adolescents as co-creators and leaders were perceived to be more successful. As such, participants recommended more meaningful engagement of young people, including young people's leadership of initiatives. Additional recommendations included the need for intersectoral engagement and a ‘whole-of-government’ approach to prevention given the complex determinants of NCD risks, and the need for evidence-based actions that are underpinned by quality data to enable monitoring of progress.

**Interpretation:**

There is a recognised need to strengthen policies and actions to address NCD risks amongst adolescents in Indonesia. Meaningful youth engagement that allows young people to take the lead, intersectoral actions, and evidence-based data driven responses were key strategies identified.

**Funding:**

UNICEF East Asia and Pacific Regional Office.

**Research in context**

**Evidence before this study**

We searched the literature for articles published from 2012 to August 2022 using terms “non-communicable diseases”, “prevention”, “risks”, and “Indonesia”; we kept search terms purposefully broad and then searched within these publications for adolescent focused studies. Our search of Ovid MEDLINE, and PubMed returned 413 titles after duplicates were removed; title, abstract and relevant full text screening identified 20 relevant publications. Searching the grey literature and reference lists revealed 5 additional publications which were relevant to our search.

We did not find any studies which explored the perceptions and experiences of stakeholders around NCD prevention. Publications were largely focused on adults with only 3 that specifically focused on adolescents. The first was an intervention in high school students to increase understanding of NCD risks using peer educators and general practitioners in collaboration with the community NCD screening program (Posbindu PTM), which was found to be successful. The investigators partnered with adolescents to develop study materials and incorporated youth empowerment as part of the intervention. A second study, from the grey literature, was our own earlier work that defined a reporting framework for NCDs and risk factors in Indonesia. That study mapped disease burden, policy, and data to highlight key NCDs of interest across the life course, and used available data to examine prevalence, geographic inequality, and data gaps. We found that greater focus on NCDs as they occur in children and adolescents, and also embedding objective measures for these age groups within existing data collection systems, would help advance actions on NCDs in Indonesia. This study had input from young people aged 18–25-years within the stakeholder consultation process. A third publication, also from the grey literature, was a research report that highlighted the need to address NCDs, with a focus on 10–24-year-olds. The authors assessed progress on NCDs in 10 countries, including Indonesia. The report recommended involving young people in the policy making process, that more data are needed including greater focus on the social determinants of health and the importance of multisectoral approaches to NCD prevention. The authors also noted that political will can be more important than a country's financial resources in its orientation to NCDs.

**Added value of this study**

This paper is the first to explore what existing strategies are in place to reduce NCD risks for adolescents in Indonesia, how young people are involved, and what is needed to advance actions in this space. A strength of this study is the way young people were prioritised throughout, with a place on the research team, participating in interviews, and formulating recommendations for action. The participants came from a broad range of stakeholder organisations and government, and were engaged in health services, program delivery, policy and strategic planning, youth advocacy, communications, monitoring and evaluation, and education. Notwithstanding the breadth of experience and backgrounds represented, clear themes emerged from the data: we report on the perceptions and experiences of those currently engaged in NCD prevention and adolescent health in Indonesia; what they identified as the strengths and limitations of programs and policies; and whether or not there was a clear focus on adolescents and young people within current programs or policies. We also provide clear recommendations for action, based on thematic analysis of the in-depth interviews, and refined by young people through a consultation workshop.

**Implications of all the available evidence**

Investment and actions to address NCDs has been fragmented and inadequate not only in Indonesia, but globally, and there is a need to rethink how we address these issues from a public health perspective. Our study is distinct in identifying how adolescents and young adults can play an important role in accelerating progress on NCDs. The key recommendations developed in consultation with participants included more meaningful engagement of young people; support for young people to take the lead; greater focus on intersectoral engagement and ‘whole-of-government’ approaches to prevention; and ensuring that evidence-based actions are underpinned by quality data.

Several aspects of our findings echo the findings in the literature. NCDs remain a significant challenge; barriers to action such as uneven policy and program implementation, poor service quality and coverage, and a lack of data continue to stymie prevention and control efforts. We also found that participants’ experiences partnering with adolescents and young people were overwhelmingly positive, with evidence of greater involvement of young people in NCD prevention in Indonesia than is reflected in the literature. This indicates the need for thorough evaluation and scaling up of implementation for those successful local partnerships between government, NGOs, and grass roots organisations.

Our findings and recommendations highlight the importance of young peoples’ leadership in driving actions on NCDs. We present clear actionable steps to improve youth engagement and to improve the current approach to addressing NCD risks in adolescents. Targeted preventative actions in this stage of life are essential to mitigate the upward trend of NCD morbidity and mortality in Indonesia.

## Introduction

Routinely collected health data in the Republic of Indonesia (Indonesia) show a rapidly changing burden of disease.^1^ While there continues to be a significant burden of communicable disease and injuries, non-communicable diseases (NCDs) have emerged as the leading causes of death and disability in the world's fourth most populous country, amongst considerable subnational inequality.^1^, ^2^, ^3^ Risks for NCDs–including high blood pressure, high BMI, high blood sugar, tobacco smoking, and poor diet–are also high in prevalence and increasing in Indonesia.^4^ Responding to this burden of NCDs is complex; it requires engagement and investment from the health sector, but also from parts of the public sector, civil society, and the commercial sector which are critical to modifying determinants, including education, agriculture, trade, transport, and social services.^5^, ^6^, ^7^ This is because prevention is key to NCD control, especially given the chronic and incurable nature of many of these diseases.

Effective prevention of NCDs requires actions across the life-course, including the developmental stage of adolescence.^8^ Adolescents (defined here as 10–24 years old) represent more than one quarter of the Indonesian population, or 67 million people,^9^ and available data show an excess burden of risk.^10^^,^^11^ Indonesian males aged 10–24 have among the highest prevalence of tobacco use globally; in 2016, it was 28.6% for males and had increased for both sexes since 1990.^12^ Daily smoking prevalence also rises steeply with age. In 2013, data from Indonesia's national health survey (RISKESDAS) showed daily smoking increased from 33% in males aged 15–19 to 60% in those aged 20–24.^13^ Having an unhealthy diet, insufficient physical activity, and obesity are also on the rise in both males and females.^10^^,^^12^^,^^14^^,^^15^ For example, the prevalence of overweight and obesity increased from 3–4% in both sexes in 1990 to 14% in females and 10.8% in males in 2016 - representing over 8 million adolescents.^10^^,^^12^ In a recent global analysis, almost half (46.5%) of Indonesian 11–17 year-olds had three or more NCD risks factors,^16^ substantially higher than in adolescents in the South-east Asia region overall (30%).^17^ Addressing risks within this important cohort is crucial, not only for adolescents' current and future health, but also for the economic and social progress of their communities.^18^^,^^19^

Globally, adolescents mostly remain at the margins of policy and prevention focused on NCDs. Dr. Marie Hauerslev (NCD Alliance board member and past-Chair of NCD Child) noted that “*We have an issue that adolescents are often seen as a healthy group; from ten onwards we often forget about health and address it when people are in their 30s, 40s and 50s and already suffering from NCDs or one or more risk factors.*”^20^^(p.9)^ There also appears to be limited adolescent engagement in research and implementation despite evidence that young people have the desire and the skills (along with the right) to be involved in issues which impact them.^21^, ^22^, ^23^ While there is some guidance on how to meaningfully engage young people,^18^ there are few examples of how youth engagement has been implemented effectively, particularly in low- and middle-income countries (LMICs). A 2021 review of youth engagement in health research found only two studies based in LMICs,^24^ both focused on sexual health.^25^^,^^26^ In Indonesia, both government and non-government sectors have increased research activities, legislation,^27^ and policy^28^ targeting NCD prevention in Indonesia. In terms of adolescent engagement, UNICEF has invested in U-report, a social messaging tool and data collection system which has been used in Indonesia to gather data on issues impacting young people, including NCDs.^29^ In Indonesia the majority of contributors (U-reporters) are aged 15–19-years.^30^ Our own literature review found only one NCD-specific Indonesian adolescent health intervention with evidence of comprehensive youth engagement.^31^

This study seeks to understand what is required to address NCD risks for adolescents in Indonesia. We explored three inter-related questions: What are stakeholders’ perspectives on existing strategies for the prevention of NCDs in Indonesia; how are adolescents currently included in these strategies; and how can adolescents be better involved to advance actions on NCDs?

## Methods

This qualitative study used semi-structured interviews to explore stakeholders’ perspectives on NCD prevention in Indonesia. Indonesia was selected by the UNICEF East Asia and Pacific regional office due to the increasing burden of NCDs, planned investments to address NCDs, and the identified need to understand how young people can be involved in these efforts.

We engaged young people in this research in three ways: we engaged an Indonesian young person as a co-researcher within the research team from the beginning of the project (ANT) to ensure the overall project and data collection was appropriately framed and ‘youth friendly’; we purposively sampled a diverse range of young people during the enquiry phase and aimed to have half of participants aged 18–25 years; and we invited young people to an additional forum to discuss the findings and co-define key recommendations.

### Development of semi-structured interviews

We developed a question guide for semi-structured interviews based on the research aims. It was then translated to Bahasa Indonesia for Bahasa language interviews (interview overview in Table 1, see full interview guide, Appendix 2). Following the first three pilot interviews (two in Bahasa, one in English), the interviewee's responses were reviewed by two interviewers to check for consistency in comprehension across languages; no major changes were required.

**Table 1:** Overview of broad concepts mapped to example interview questions.

Overview	Example Questions
Understanding NCD risks among adolescents in Indonesia.	What do you know about the risk factors associated with NCDs? How important are these risks in adolescents?
Existing policies and programs focused on NCDs or adolescent health.	Do you know of any current initiatives or programs being run which are particularly focused on NCD prevention? Were any of these initiatives or programs led by a youth-run organisation or network?
Strengths and challenges in current policy or programming.	What gaps do you see in current adolescent health and well-being initiatives, policies, or programs? Do you know of any programs which focus on training or capacity building of adolescents?
Stakeholders, actors, and organisations active in NCDs or adolescent health.	Who are your key partners on adolescent health and NCD-focused initiatives? Which platforms do you know of that are currently used to reach and engage with adolescents?
Perceptions and experiences of young people.	Have you ever received any training, mentoring, or support that has helped you? Where do you feel you can have the biggest impact (family, school, community, peers, friends)?
Perspectives on a youth led approach.	What do you think about a youth-led approach to NCD prevention in Indonesia?

See Appendix 2 for full interview guide.

### Participants

Stakeholder organisations were identified from an earlier mapping of key NCD actors in Indonesia^13^^,^^32^ supplemented by advice from the UNICEF Indonesian country office. Stakeholder organisations included policymakers and service providers in government (national, provincial, and district), local and international Non-Government Organisations (NGO), and local NCD- or youth-focused organisations. We invited those who had experience working in diverse settings (diverse geography or with vulnerable populations) and we sought a gender balance of key informants. We prioritised participants aged 18–25 years because we wanted to speak to young people as stakeholders, both due to their age and as practitioners and leaders in relevant fields. Being over 18, they were able to give informed consent for participation.

Stakeholder organisations were also offered the opportunity to refer 18–25-year-olds from their organisation who might be interested in taking part. We made it clear that the research team would not report back to organisations whether the young person had decided to take part. Those referred were told their decision to take part would be kept confidential from their referrer (see participant consent form, Appendix 3). There were no incentives or reimbursements offered to participants. Given the earlier engagement with some stakeholders,^13^ one interviewer (KC) had consulted with some participants previously. Box 1 details the inclusion and exclusion criteria. Key informants could have experience in adolescent health, adolescent development, or NCDs. However, while the details varied between participants, most had experience across both adolescent health or advocacy and NCDs, such as an NGO employee who worked across multiple projects in adolescent health including some that focused on NCDs.

Box 1 **Participant inclusion and exclusion criteria.**

Inclusion criteria:

• Key informants from selected stakeholder organisations: working or volunteering in policy, programming, implementation, or advocacy, focused on adolescent health, adolescent development, or NCDs.
• Aged 18 years or over.
• Able to provide voluntary informed consent.

Exclusion criteria:

• Children or adolescents aged less than 18 years.

### Procedure

Potential participants were invited via email to take part in an individual interview. The initial email advised that interviews could be conducted in Bahasa or English following written consent prior to participation. Verbal assent was further obtained at the interview and participants were reminded of their ability to withdraw at any time and that they could skip any question if desired. Of the 28 people approached via email, 21 (75%) agreed to take part and none withdrew post consent.

Due to the COVID-19 pandemic, interviews were conducted online using Zoom videoconferencing (Zoom Video Communications, Inc) between 6th April 2021 and 25th May 2021. Participants were advised to join the videoconference from a private space of their choosing. Interviews were led by one of two female post-graduate qualified researchers, who were either English-speaking (KC) or Bahasa-speaking (NW). Six of the Bahasa-language interviews were also attended by a young Indonesian graduate researcher (ANT), who participated in planning the interviews, note-taking, asking questions from the interview guide, translation, and transcribing. Videoconference software provided separate audio and video recordings of the interviews; video recordings were deleted and audio recordings were retained for transcribing and translation (as outlined in the participant consent forms, Appendix 3). Transcripts were not returned to participants for comment. All quotes included in the findings and recommendations reported here are from these 21 interviews.

### Analysis

Audio recordings of interviews were transcribed and translated into English where required. Transcripts were thematically analysed by researchers using a pre-defined framework based broadly on the research aims.^33^ Using NVivo12, transcripts were coded, and relevant quotes were mapped to the framework. Two researchers crosschecked and discussed the transcripts, data saturation, and the themes arising from the interviews. Two researchers and the lead investigator discussed and reached consensus on the findings, selected relevant quotes, and drafted recommendations arising from the interviews.

Initial findings were disseminated to all participants and the broader research team for validation and feedback. Two online workshops were held over videoconference by the lead researchers, one for UNICEF Indonesia staff and the research team, the other specific to youth participants (18–25 years). Both workshops had a short presentation of findings and draft recommendations which supplemented a written report that had been previously circulated. In the UNICEF and research team workshop, feedback was given, and the findings discussed. In the second workshop seven young people attended (2 male, 5 female), with three researchers, one of whom provided translation between English and Bahasa. The young people who attended represented Indonesian NGOs, International NGOs, the UN sector, and youth-focused organisations. Following the presentation participants were asked to consider three questions to guide the discussion (see Appendix 4). The youth participants who attended had a pivotal role in helping to formulate and refine the final recommendations and suggested actions arising from this research. Other participants (>25 years and young people who could not attend the workshops) had the opportunity to provide written or verbal feedback (via telephone); five people provided feedback by email.

### Ethics

This study received ethical approval from the Alfred Hospital Ethics Committee, Melbourne, Australia (approval 754/20) and the Health Research Ethics Committee of the Faculty of Public Health, Universitas Hasanuddin (1305/UN4.14.1/TP.02.02/2021).

### Role of funding source

This study was funded by UNICEF East Asia and Pacific Regional Office (EAPRO). The overarching aims of the study were guided by the funding brief from UNICEF EAPRO, but UNICEF was not involved in data collection, analysis or drafting the key recommendations. UNICEF Indonesia staff were consulted on the draft recommendations, and their feedback was considered by the lead investigators (KC, PA, AA) and young people in framing the final recommendations. None of the interviewers or interviewees were employees or volunteers at UNICEF. All authors had access to the data in the study, approved the final manuscript and were responsible for the decision to submit the manuscript.

## Results

We interviewed 21 stakeholders (Table 2). All included quotes are from the individual stakeholder interviews. Quotes are identified by sector type, and quotes attributed to young people are noted as ‘YP’. Omitted text in a quote is indicated as ‘…’. Participants have been categorised into the following sector types for the purpose of clarity within the results, without identifying the individual participant. ‘Public Sector’ referred to those holding roles within central, provincial, and district government in policy or health services delivery; ‘Indonesian NGO’ referred to stakeholders from Indonesian non-government organisations that have a broad development agenda; ‘International NGO’ were those from international organisations with similarly broad agendas, including UN sector organisations; ‘Youth-focused/NCD-focused organisation’ were either youth-focused or single health issue focused organisations or advocacy groups.

**Table 2:** Participant characteristics.

	N = 21
Gender, female n (%)	13 (61.9)
Age, 18–25 years n (%)	9 (42.9)
Sector type n (%)	
Public sector	4 (19)
Indonesian NGO	4 (19)
International NGO	5 (23.8)
Youth-focused/NCD-focused organisation	8 (38.1)
Location, n (%)	
Jakarta	9 (42.9)
Other provinces	12 (57.1)
Interview language, Bahasa n (%)	8 (38.1)
Interview length in hours, median (range)	1.5 (1–2.5)

NCDs and risk factors were considered important health issues impacting all ages and populations across Indonesia. Participants described the link between individual behaviours and NCDs. Social determinants as underlying drivers of risks were acknowledged, as was the influence of social norms, gender norms, and societal expectations.

### Current strategies to address NCD risks in adolescents in Indonesia

Participants identified strategies that targeted education, youth empowerment, health promotion, and NCD screening, that were developed by government (usually within the Ministry of Health), NGOs, and grassroots or youth-led organisations. Strategies relevant to adolescents are outlined in Table 3. The Ministry of Health was a key partner to most stakeholders. Participants recognised that many of the existing primary prevention and screening activities were focused on adults and were insufficient in their current orientation to address NCDs and their risk factors in adolescents. Current strategies aimed at adolescents for NCDs and risk factor prevention were limited in their effectiveness due to inconsistent implementation, narrow coverage, and lack of clear policy targets. There were however some examples of successful education and health promotion programs focused on adolescents that were developed by NGOs and grassroots or youth organisations. Broadly, participants highlighted that youth engagement contributed significantly to successful implementation of programs and services, particularly at the grass roots level. However, in the policy and regulation space, participants noted a lack of meaningful engagement.

**Table 3:** Adolescent relevant prevention focused strategies, stakeholders, and example programs identified.

Description of action or program	Implementation	NCD focus	Adolescent focus	Target population
**Peer educator or peer support programs.** Adolescents are supported to promote health-enhancing change among their peers. We found examples from Ministry of Health (MoH), Plan International Indonesia, Into the Light, Lentera Anak, and Centre for Indonesia's Strategic Development Initiatives (CISDI).				
Young Health Programme (YHP) Peer network: YHP is an NCD prevention initiative for Adolescents.	Yayasan Plan Indonesia. Web: plan-international.or.id	Yes, NCD prevention.	Yes, designed for and with adolescents.	Adolescents in selected cities.
**Digital media campaigns** held on social media, online platforms, sometimes in partnership with MoH, or facilitated through the arts (exhibitions or installations). We found examples from Vital Strategies, Indonesia Adolescent Health Association (AKAR), CISDI, and Into the Light.				
Show Your TRUE Colours media campaign: Vital Strategies partners with a village overrun by tobacco branding to reclaim and re-brand their village.	Vital Strategies. Web: vitalstrategies.org/tobacco-control-case-study-indonesia	Yes, Tobacco. They have several programs focused on NCD prevention.	Campaign engaged a whole village, including adolescents.	Whole of population.
AKAR Indonesia: runs digital media campaigns on Instagram on adolescent health.	Indonesian Adolescent Health Association. Instagram @akar_inaha	Yes, adolescent health focus but often includes NCD content.	Yes, designed for and with adolescents.	Adolescents and those who work with them.
**Youth forums, meetings, or roundtables** where young people give their opinions on issues important to them. Examples include Youth Town Hall (a World Health Organisation, MoH, and CISDI collaboration), Forum Anak Desa, and AKAR youth forum.				
Forum for Young Indonesians: a platform for youth to discuss relevant development and social issues.	Centre for Indonesia's Strategic Development Initiatives. Instagram @fyindonesians, Web: cisdi.org	Yes, often has an NCD focus like campaigning for a sugar-sweetened drinks tax.	Yes, designed for and with adolescents.	Adolescents and young people.
U-report: a social messaging tool and data collection system which collects user's opinions on health issues and to send public health messaging.	UNICEF. Web: indonesia.ureport.in	Somewhat. Adolescent health focus but often includes NCDs.	Yes, designed for and with adolescents.	Adolescents and young people.
**Youth empowerment/ advocacy courses.** Young people receive capacity building for particular issues or skills. We found examples from The Leader, Red Cross Youth, Into the Light, CISDI, Smoke Free Agents, Vital Strategies.				
Dreammaker training: Youth empowerment and capacity building in Banda Aceh. The Leader also runs programs throughout Indonesia.	Implemented by The Leader a volunteer-led youth association. Web: the-leader.org	Somewhat. Youth-led so the focus varies, but often includes NCDs.	Yes, designed for and with adolescents.	Adolescents and young people.
Rise and Shine: capacity building for young people on mental health and suicide prevention. This is for Into the Light volunteers, however they also run public facing education.	Into the Light, a youth-based community. Instagram @intothelightid, Web: intothelightid.org	Yes, focused on mental health and suicide prevention.	Yes, designed for young people who aspire to be mental health advocates.	18–25-year-olds in Jakarta, Bandung, and Yogyakarta.
**Education or health promotion programs.** Many organisations have implemented health education programs and continue to do so. These are often focused on issues impacting a particular group or community, or a single issue such as suicide prevention. We found examples from Lentera Anak, 9 cm (Global No Cigarette Movement), AKAR, The Leader, Red Cross Youth, Into the Light, CISDI, Smoke Free Agents, Vital Strategies, Ministry of Education (MoE), and MoH.				
9 cm (Global No Cigarette Movement) run digital campaigns, school outreach, and educational programs.	9 cm (Global No Cigarette Movement). Instagram @9cmindonesia	Yes, tobacco control, but also other issues such as healthy environment.	Yes, designed for and with adolescents and young people.	High-school aged adolescents.
GERMAS: a health promotion initiative designed to encourage people to live healthy and have an active lifestyle.	Indonesian MoH, across multiple levels of government. Instagram @kemenkes_ri, Web: kemkes.go.id	Yes, e.g., Piring Makanku (My Plate) is a nutrition program within GERMAS.	Not specifically adolescent focused or led.	Whole of population
CERDIK: A health promotion initiative for prevention of NCDs.	Indonesian MoH, across multiple levels of government.	Yes. CERDIK is an initiative to prevent NCDs.	Not specifically adolescent focused or led.	Whole of population.
**Screening and counselling programs.** Young people are screened at health centres, schools, or community events and then provided with health education or offered a specific intervention. We found these programs were often implemented through existing health services overseen by MoH.				
Usaha Kesehatan Sekolah (school health service): has three pillars, health education, health services in school, and healthy school environment.	Indonesian MoE together with 3 other ministries, MoH, Religious Affairs, and Home Affairs.	Somewhat, amongst other health issues.	School student focus, some activities may be adolescent led.	School students.
Pos Pembinaan Terpadu untuk Penyakit Tidak Menular (Posbindu PTM): Integrated health post for preventing NCDs.	Posbindu PTM can be integrated into community health centres or can ‘pop up’ in schools, workplaces, or communities.	Yes, NCD screening, education, and referral to health services.	Some youth-focused initiatives in partnership with local groups, implementation varies.	Ages 15+.
Posyandu Remaja (PR): adolescent health outpost, designed to increase engagement with health services.	Typically Puskesmas staff, with village level implementation supported by local volunteers.	Yes, strong NCD focus, amongst other adolescent health issues.	Adolescent focused, and often adolescent led, implementation varies.	Adolescents and young people.
Pelayanan Kesehatan Peduli Remaja (PKPR): adolescent friendly health service includes education, clinical services, peer educator training, and counselling.	Puskesmas community health centres and owned by local government health agencies.	Somewhat, amongst other adolescent health issues.	Adolescent focus, unclear if adolescent led.	Adolescents and young people.
Sehat Tanpa Asap Rokok, healthy without smoking (SEATAP): School health staff refer students who smoke to Puskesmas for quit counselling and training as peer educators on smoking risks.	Puskesmas Health promotion team in Makassar. SEATAP is a broad national program, implementation varies.	Yes, focused on smoking prevention and cessation.	Secondary student focus here, but SEATAP program varies by location.	Students in specific districts.

#### Current programs and services

Participants reported that existing NCD focused screening and health promotion programs were adult focused. For example, Posbindu PTM, the government's NCD screening program, although open to adolescents from age 15, are not orientated towards this age group. Participants felt that current adolescent health initiatives did not sufficiently focus on NCDs.

*“When it comes to adolescents in terms of health the focus is always SRH (sexual reproductive health) or infectious disease – it’s not tobacco or obesity.”*

*(Public sector)*

However, Posyandu Remaja (adolescent health outpost) and Pelayanan Kesehatan Peduli Remaja (PKPR; adolescent friendly health services) were both identified as adolescent services which had some NCD focused activities. Examples of these activities included an adolescent smoking cessation program at PKPR, co-led with a local youth-focused organisation, and NCD screening events held at Posyandu Remaja organised and facilitated by local adolescents, to identify those at risk (measuring height, weight, blood pressure).

*“Posyandu Remaja is actually one of the success stories of youth engagement at the grassroots level – it is a bottom-up program – initiated by communities in East Java which has already spread to many provinces, yet the quality of the implementation varies”*

*(Youth-focused org)*

Participants expressed that these two services had great potential to meet the needs of adolescents and fill an important gap in prevention activities. They provided examples of partnerships between Posbindu and a local university, and PKPR with local NGOs, which they described as successful. Importantly, participants felt that the success of these examples was due to adolescents’ active involvement and meaningful engagement in these activities. Some believed that Posbindu activities should be extended to a younger age, while others felt that PKPR and Posyandu Remaja were the better forum for adolescent focused activities. However, regardless of which service participants felt should be extended, they highlighted the need for better monitoring and evaluation of implementation to ensure an evidence-based approach to service provision. While program-level evaluation was regularly built into local services, participants said it was not possible to evaluate outcomes against national targets and indicators.

#### Current policy and regulation

Participants identified various government policies and regulations which they felt were designed to control NCDs. However, they felt that efforts were hampered in two important ways: lack of enforcement of key regulations, and lack of meaningful youth engagement in the policy-making process.

Participants noted that regulations around food advertisement and labelling, tobacco advertising and sponsorship, tobacco packaging and labelling, and the enforcement of smoke-free places were not well implemented.

*“...Food labelling is often covered by promotional messages.”*

*(Indonesian NGO)*

*“I am aware of the PP 109 (tobacco control). I think it says do not sell the cigarettes to children, but in reality, the shops are selling cigarettes to children or adolescents. There is no punishment or sanction if they are doing this.”*

*(YP, Indonesian NGO)*

*“Kawasan Tanpa Rokok (KTR, Non-smoking area): However, the implementation is not going well. Even though there are plenty of signs in public to not smoke in the specific designated areas but there are still people smoke in there. For sanctions on that regulation, it still has to be strengthened.”*

*(Public Sector)*

While issues such as sanctions are key to enforcement, we also found that participants wanted to support greater involvement of adolescents in the policy-making process. In the past, participants felt this work had been largely tokenistic.

*“We want more for youth than just come and talking to the Minister, we want to stay involved in the policy setting and in policy publications as well. We don't have enough of that kind of platform… voicing their needs and making the regulations. As I mentioned before a lot of regulations are not designed to protect our youth...”*

*(Indonesian NGO)*

*“We were told we (the youth) were being invited to discuss about some policies… at the event it was already drafted, and we were just revising and reading them… it was pre-made by the government and then it was given to the youth to just review and that was really disappointing - we really thought that we were going to be a part of this policy.”*

*(YP, Youth-focused org)*

*“I think one of the most important things to consider is to involve young people from early… if the government or UNICEF would like to initiate new activities that are targeted to young people, they should listen to young people”*

*(YP, Youth-focused org)*

### How are adolescents currently included in strategies to address NCDs?

Participants reported many examples of adolescents on the front-line of NCD prevention initiatives in Indonesia: in peer-to-peer programs, advocacy, education, and community actions, where they were reported to be leaders, project coordinators, program and evaluation officers, health professionals, national coordinators, and student volunteers. Participants felt that when adolescents were involved, they contributed unique ideas and energy to programs and initiatives, they were skilled in communicating with and engaging other adolescents, and very aware of the issues impacting their cohort.

*“Listen to them! They have brilliant ideas… young people are underestimated.”*

*(International NGO)*

*“They [young people] have a style we don't understand, but the best part is that they can communicate effectively with their age group when we can’t.”*

*(Indonesian NGO)*

Youth engagement was seen by many participants as both the strength of existing strategies and the best opportunity for driving further actions. However, participants described varying experiences of collaborating with government and other organisations. It appeared that government departments were more difficult to engage with than non-government organisations.

*“A positive with youth led orgs and UN agencies - they are really interested in what we are saying. With government organisations its harder to do - it’s harder to convey our opinions to them.”*

*(YP, NCD-focused org)*

*“I prefer to work with the research division (of Ministry of Health) to be honest, because they work in partnership with the Universities, who are well informed… So, they really listen to us, about what kind of questions we want to address, as patients, as young people, and on how to deliver the program and disseminate the research results.”*

*(NCD and youth-focused org)*

*“Our journey doing advocacy wasn't easy - the government is not always cooperative working with young people - as we know there's a social hierarchy in our society.”*

*(YP, Youth-focused org)*

Young participants also described a lack of follow-up on issues raised and an absence of transparency on how their engagement was applied, leaving many with the perception that their work was tokenistic.

*“There is no accountability for what we tell them... how are young people's opinions being heard in the government? We can reach out and speak to them, but we don't know what they do with the information.”*

*(YP, Indonesian NGO)*

*“We often are not equal... We don’t get feedback from them. We need a clear framework from the beginning.”*

*(YP, NCD and youth-focused org)*

### What are the recommendations to drive action?

Draft recommendations were based on the findings from analysing 21 stakeholder interviews. The final key recommendations (Box 2) were then co-developed with young people and the research team, who identified specific areas in which adolescents could take the lead and promote actions on NCDs. Quotes were drawn from the stakeholder interviews, not the subsequent discussions to refine the recommendations.

Box 2 **Key recommendations.**

1. Meaningfully engage young people as equal partners in the response to NCDs.
2. Identify and support areas where young people can take the lead.
3. Prioritise intersectoral engagement for action on NCDs.
4. Facilitate evidence-based actions and policy, based on sound data.

#### Meaningful engagement with young people

Most participants saw meaningful youth engagement as crucial for driving actions on NCDs in Indonesia. However, for youth engagement to avoid the pitfalls of tokenism, many participants cited the need for a framework (a set of priorities and guidelines) to govern youth engagement with mutually agreed expectations and outcomes. Participants also felt that there was a need to develop the capacity of policy makers and stakeholders to work more effectively with adolescents. Additionally, they urged steps be taken to ensure that engagement was representative of the diversity of adolescents in Indonesia.

*“A priority action is to create a roadmap for youth engagement with the government, a framework … what do they envision that youth will be engaged in? I don't think that kind of discussion has happened internally in government.”*

*(YP, Indonesian NGO)*

*“Link youth organisations with (government), make safeguarding principles together… have an open and transparent way of doing things, like deciding who gets to be involved in what.”*

*(International NGO)*

*“So, before we include more people the government needs to improve how they engage.”*

*(YP, NCD-focused org)*

Those who had developed such frameworks previously found they had facilitated quality engagement with youth.

*“First thing is that we made our own guidelines of youth involvement and engagement. So that we as the members of that council knew our rights… Even before you ask the youth of their opinions, start with what kind of expectations you have, or what do you think is your right to be heard, because it will really affect any other activities after that… but make sure that everyone understands how to meaningfully engage young people.”*

*(YP, NCD-focused org)*

Participants felt that another step towards meaningful engagement was to ensure that youth engagement is representative of diversity (economic, geographic, gender, and ethnicity), noting that such efforts must be adequately resourced and explicitly defined. Some felt that there was a risk of bias if only particular groups were consulted.

*“But even in Indonesia that claims it's country as a very diverse country, but yet we do not have such representation… LGBTQI, indigenous, ethnic minorities. I'm trying to tell them that young people... we aren't only people who live in Java and, you know, a certain religion and ethnicity background, no… it’s also us who are not included in (those) categories.”*

*(NCD and youth-focused org)*

*“We need to offer routes for young people to get involved who are usually marginalised… there is a risk when a big international organisation is involved, that group (of young people) you’re talking to becomes elite or exclusive, just an extension of the bureaucracy.”*

*(International NGO)*

#### Supporting young people to take the lead

Participants identified four specific ways that young people can take the lead with the support of other stakeholders.

1. Young people can raise awareness of NCDs and their risk factors within their families, peer groups and communities.
2. Young people can support other young people and amplify their voices.
3. Young people can lead campaigns for the inclusion of youth in policy.
4. Young people can advocate for the transparent and accountable implementation of laws, regulations, and policies.

Participants felt a crucial part of allowing young people to take the lead is providing support structures to enable them. One action suggested by several participants was to promote connections between youth initiatives and leaders (government or non-government): creating intergenerational partnerships. There was a common interest from young participants to be more connected to both mentors and other stakeholder networks.

*“We try to foster collaboration between initiatives… Without connections they (young people) are trying to solve problems on their own.”*

*(YP, Indonesian NGO)*

*“I hope we could receive more supports that are maybe in the form of networks or connections to the right people.”*

*(YP, Indonesian NGO)*

Participants shared some examples of how these intergenerational partnerships had specifically helped them to engage in meaningful projects.

*“Some people that I know, they make the effort to give me a seat at the table”*

*(YP, NCD focused org)*

*“I thought I was just studying a bachelor and didn't have enough knowledge or experience in this field, but my co-workers (adults) always encouraged us to speak up. For example, giving us opportunities in the meetings. I have overcome it and I have now become more confident talking in public including to stakeholders from the government.”*

*(YP, Youth-focused org)*

It was also acknowledged that those young people who advocate in public forums need additional support, as this kind of advocacy was understood to carry an element of risk.

*“Young people have been pressured by industry after working with us… young people being bullied on social media and other platforms; we need groups of young people that support each other.”*

*(International NGO)*

#### Improved intersectoral actions

Participants identified effective intersectoral actions as central to progress in Indonesia. This included facilitating interactions between different ministries within government, across the various levels of government, and government funded service providers.

*“The difficulty is a siloed approach when we talk about young people. Ministry of Finance, Ministry of Industry, other ministries need to be involved - they can't all be pretending nothing is happening or that they're not involved.”*

*(Public sector)*

Stakeholders felt that better intersectoral engagement was needed to improve enforcement, regulation, and implementation of laws, particularly those focused on tobacco control and advertising, and food labelling and advertising. To improve, participants felt that intersectoral coordination needed to extend beyond government and across communities, schools, businesses, the arts, agriculture, and policy makers.

#### Address data gaps to improve accountability

Participants identified the need for high quality data to enable transparency and accountability in three areas: firstly, for priority setting – to understand the health needs of the adolescent population; secondly, for advocacy – to drive changes in legislation, programming, and funding; and thirdly, for monitoring and evaluation. Participants emphasised the role of data for better monitoring and evaluation, both for their own work and to build accountability and trust in government-funded programs. Participants were clear that they felt they did not have access to adequately disaggregated epidemiological data for the local communities their programs were focused on.

*“We don't even have any data on... inequality, on gaps, illness gaps between different ethnic groups, or sexual orientation - we don't have such data, we don't have such research.”*

*(NCD and youth-focused org)*

Several participants acknowledged the existence of current data systems in Indonesia which have some relevant coverage for adolescents and NCDs, such as RISKESDAS survey data, and data generated from screening activities in Posbindu and Posyandu Remaja. However, they were unsure how to access the data or receive the support to meaningfully use it.

## Discussion

We found that almost all existing national NCD strategies target adults. Furthermore, most current health services and programs for adolescents do not routinely address NCD risks. The few that have both an adolescent and NCD focus are either smaller targeted programs or have insufficient quality data to monitor and evaluate their effectiveness. This includes those programs that were perceived to be the most successful, such as Posyandu Remaja. We found that efforts to address NCDs are beginning to be embedded in data systems (in surveillance and outcome monitoring), and in policies and regulations with an NCD focus.^1^^,^^3^^,^^27^^,^^28^^,^^34^ However, existing policies lack specific targets or measurable indicators for adolescents, and regulations which could target key risks (such as PP109, or KTR - non-smoking areas) were broadly perceived as poorly enforced. Although Indonesian young people already carry a variety of roles in NCD prevention (as peer-to-peer educators and adolescent cadres, in grassroots organisations or in professional service provision roles), there was strong support for much greater youth involvement in higher-level priority setting and policy.

Our study is distinct in identifying that adolescents and young adults are a major resource to drive progress on NCDs in Indonesia. We have shown that stakeholder representatives from a variety of backgrounds and roles identified meaningful youth engagement as both vital to current achievements and the best opportunity for future success in NCD prevention in Indonesia. The themes which arose as priority actions to ensure meaningful youth engagement resonate with those from other studies. Actions such as developing standards and guidelines for engagement, transparency in monitoring, evaluation, and dissemination, and supporting and strengthening partnerships involving young people, are consistent with a recent global stakeholder consultation on how to improve youth engagement in health research.^21^^,^^35^ Another global report on engaging young people in health and sustainable development found that stakeholders highlighted the importance of engaging those from diverse backgrounds, allowing them to lead, and the need for capacity strengthening within organisations to have meaningful engagement with young people.^23^ Indonesia is not the only country in the region experiencing difficulties making progress on NCDs. A recent assessment of progress in seven south-east-Asian countries (including Indonesia) and implementation of WHO ‘best buy’ interventions for NCDs identified that scarcity of standardised monitoring and evaluation processes, gaps in institutional capacity, limited funding, and lack of intersectoral actions contributed to the insufficient progress on reducing NCDs in the region.^36^

Another theme underpinning our findings was the need for greater transparency and accountability in NCD prevention and control. Improved accountability in implementation, monitoring, youth engagement, communication, and governance is likely to improve priority setting, contribute to better opportunities for intergenerational partnerships, and ultimately accelerate progress. This need is not new to global health or the NCD agenda,^37^^,^^38^ and several participatory frameworks have been designed to improve accountability. Patton and colleagues adapted one such accountability framework for adolescent health, which combines a four-step process of governance, action, assessment, and communication, with (crucially) independent oversight and youth engagement at the centre.^18^^,^^39^ This framework provides a basis from which policymakers, service providers, and other organisations can tackle the key recommendations of this study, while keeping a focus on accountability.

The Indonesian Ministry of Health's current action plan for the directorate for prevention and disease control (2020–2024) has only one NCD target that includes adolescents, the ‘early detection screening for NCD risks including people aged 15 and above’, despite the focus on prevention within the action plan generally. The absence of policy targets highlights that young people are not being adequately considered in the context of the significant NCD burden in Indonesia. The reported success of grassroots services like Posyandu Remaja is promising, given the lack of relevant policy focus. With strengthened implementation and evaluation there is a real potential to meet adolescent needs for screening and early intervention of NCD risks. Posbindu PTM is the government's NCD screening program which is mandated to screen for NCDs in people over 15, however due to resourcing and access issues it has not been well utilised by adolescents.^40^ There are examples of innovative collaborations between government and NGOs designed to improve uptake amongst adolescents. One study used peer-to-peer educators to complement the Posbindu activities in partnership with local GPs; a great example of how adolescents can be meaningfully engaged within local partnerships.^31^ Ideally, given Indonesia's decentralised system of government and resource constraints, innovative funding to improve these services could be flexible to allow communities to promote links across government services (such as Posyandu Remaja, PKPR, and Posbindu) and beyond. This could encourage youth engagement as a tool to find a solution which works within local constraints and provide incentives for intersectoral collaborations. To properly monitor and evaluate such intersectoral activities, clear policy targets are needed, as well as indicators with which to measure success, and data to inform the indicators.

The Indonesian government has invested heavily in health data collection, for example, Posbindu PTM (screening for NCDs), the sample registration system, and the national health survey.^3^^,^^8^^,^^27^ However, issues with quality (incomplete coverage, unvalidated measures) and availability (data accessibility difficulties between ministries, levels of government, and beyond) continue to hamper evidence-based action on NCDs. To apply an equity lens to NCD action and monitor impacts sub-nationally and for marginalised populations, better access to disaggregated data is needed. One issue is that key data on social determinants and risk factors are held across ministries such as the Ministry of National Development Planning (Bappenas) data repository, air quality monitoring data, health facilities research (RIFASKES), and the national socio-economic survey (SUSENAS). The dissolution of the Ministry of Health research division (formerly Balitbangkes) and integration of the recently created Health Research Organization (Organisasi Riset Kesehatan) under the umbrella of the National Research and Innovation Agency (Badan Riset dan Inovasi Nasional, BRIN) has the potential to promote research on NCDs, facilitate data access across health datasets and beyond, which could foster inter-sectoral research collaborations. Advocacy from adolescents could play an important part to ensure that the policy focus on prevention is reflected in data collection systems that include adolescents, and that the data collected is accessible to support evidence-based priority setting in adolescents.

A strength of this study is that young people's voices were prioritised throughout. We had a female Indonesian young person on the research team, young people participated in the interviews, and young people also helped to define the recommended actions. The young participants were all working in some aspect of NCD prevention, adolescent health, or youth advocacy which is not only a mechanism from which the recommendations are contextually grounded, but also helps ensure that there is alignment with the needs of those who wish to translate this research into action. Another strength of this study is the breadth of expertise represented in the interviewees. We spoke to people engaged in health services, program delivery, policy, strategic-planning, youth advocacy, communications, evaluation, and education. What we gained in breadth of experience was lost in depth of detail from specific sectors, which could be viewed as a limitation of the study. However, despite the variety of roles and responsibilities of our participants, clear themes emerged across perspectives that ranged from student volunteers through to national directors. This commonality indicates that the recommendations to drive action in partnership with young people could be broadly useful. Another potential limitation to the study is that the findings may not be widely generalisable beyond Indonesia. However, many of the issues and recommendations discussed by our participants are consistent with other global and regional studies.^21^^,^^23^^,^^35^^,^^36^

In conclusion, our study found universal support amongst a diverse group of stakeholder representatives for greater youth engagement in the prevention and control of NCDs in Indonesia. In fact, young people's involvement in policy and implementation was seen as the key to current and future success, and young participants in our study articulated that further steps are needed to ensure meaningful youth engagement. Arguably, finding pathways to amplify adolescent voices and allowing them to take the lead will help to give greater priority to NCD prevention within government and non-government organisations alike, improve intersectoral engagement, and help address critical data gaps.

## Contributors

KC and PA conceptualised this study. KC, PA, and AA developed the study protocol. KC, PA, and NW developed the data collection tools, with expert feedback from AA and ANT. KC, PA, NW, AA, ANT, AC, GS supported implementation of the study, with interviews led by KC, and NW, and assisted by ANT and AA, and validation workshops held by KC, AA, and PA. NW & ANT handled all translation of transcripts in Bahasa Indonesia to English. KC, IA, LL, and DAP completed the literature review. KC, PA, and NW led the analysis. Specific expertise for this study was sought from SMP, AC, SAE, GS, AA, ANT, DAP (the Indonesian context); ANT, IA, LL, DAP (youth perspective and engagement); KF (data availability and interpretation), DD, SS, GP (adolescent health and wellbeing), and all authors contributed to the interpretation and drafting of the manuscript. All authors approved the final manuscript and were responsible for the decision to submit the manuscript.

## Data sharing statement

Qualitative transcripts are not available for sharing due to the risk of identifying individuals. De-identified data may be shared on a case-by-case basis upon requests to the corresponding author.

## Declaration of interests

Researchers from Murdoch Children's Research Institute are supported by the Victorian Government's Operational Infrastructure Support Program.

KC is supported by a postgraduate scholarship from Australia's National Health and Medical Research Council (NHMRC) GNT1190911. KC & DD are supported by The Centre of Research Excellence in Driving Global Investment in Adolescent Health funded by NHMRC GNT1171981. DD is supported by an NHMRC Early Career Fellowship GNT1162166 (2019-2022), and by an ARC Discovery Early Career Award DE230101174 (2023-2025). PSA is supported by a NHMRC Investigator grant GNT 2008574.

The authors declare no conflict of interests.

## References

[1] Ministry of Health of the Republic of Indonesia (2019). http://labdata.litbang.kemkes.go.id/images/download/laporan/RKD/2018/Laporan_Nasional_RKD2018_FINAL.pdf.

[2] Mboi N., Surbakti I.M., Trihandini I. (2018). On the road to universal health care in Indonesia, 1990-2016: a systematic analysis for the Global Burden of Disease Study 2016. Lancet.

[3] World Health Organization (2017).

[4] Mboi N., Syailendrawati R., Ostroff S.M. (2022). The state of health in Indonesia's provinces, 1990-2019: a systematic analysis for the Global Burden of Disease Study 2019. Lancet Global Health.

[5] Hosseinpoor A.R., Bergen N., Mendis S. (2012). Socioeconomic inequality in the prevalence of noncommunicable diseases in low- and middle-income countries: results from the World Health Survey. BMC Public Health.

[6] Marmot M., Bell R. (2019). Social determinants and non-communicable diseases: time for integrated action. BMJ.

[7] Gilmore A.B., Fabbri A., Baum F. (2023). Defining and conceptualising the commercial determinants of health. Lancet.

[8] Schroders J., Wall S., Hakimi M. (2017). How is Indonesia coping with its epidemic of chronic noncommunicable diseases? A systematic review with meta-analysis. PLoS One.

[9] Global Burden of Disease Collaborative Network (2020).

[10] Global Burden of Disease Collaborative Network (2020).

[11] UNICEF (2021). https://data.unicef.org/resources/adolescent-health-dashboards-country-profiles/.

[12] Azzopardi P.S., Hearps S.J.C., Francis K.L. (2019). Progress in adolescent health and wellbeing: tracking 12 headline indicators for 195 countries and territories, 1990-2016. Lancet.

[13] Cini K., Ansariadi A., Sawyer S., Azzopardi P. (2019).

[14] Rachmi C.N., Jusril H., Ariawan I., Beal T., Sutrisna A. (2021). Eating behaviour of Indonesian adolescents: a systematic review of the literature. Public Health Nutr.

[15] Rachmi C.N., Li M., Alison Baur L. (2017). Overweight and obesity in Indonesia: prevalence and risk factors-a literature review. Public Health.

[16] Pengpid S., Peltzer K. (2019). Behavioral risk factors of non-communicable diseases among A nationally representative sample of school-going adolescents in Indonesia. Int J Gen Med.

[17] Uddin R., Lee E.Y., Khan S.R., Tremblay M.S., Khan A. (2020). Clustering of lifestyle risk factors for non-communicable diseases in 304,779 adolescents from 89 countries: a global perspective. Prev Med.

[18] Patton G.C., Sawyer S.M., Santelli J.S. (2016). Our future: a Lancet commission on adolescent health and wellbeing. Lancet.

[19] Sawyer S.M., Afifi R.A., Bearinger L.H. (2012). Adolescence: a foundation for future health. Lancet.

[20] Chipman A., Koehring M. (2019). Addressing non-communicable diseases in adolescence.

[21] Wilson O., Daxenberger L., Dieudonne L. (2020).

[22] Diers J. (2013). Why the world needs to get serious about adolescents: a view from UNICEF. J Res Adolesc.

[23] Bulc B., Al-Wahdani B., Bustreo F. (2019). Urgency for transformation: youth engagement in global health. Lancet Glob Health.

[24] Sellars E., Pavarini G., Michelson D., Creswell C., Fazel M. (2021). Young people's advisory groups in health research: scoping review and mapping of practices. Arch Dis Child.

[25] Wolf H.T., Davidoff K., Auerswald C.L. (2019). Health care experiences of youth living with HIV who were lost to follow-up in western Kenya. J Assoc Nurses AIDS Care.

[26] Reynolds C., Sutherland M.A., Palacios I. (2019). Exploring the use of technology for sexual health risk-reduction among ecuadorean adolescents. Ann Glob Health.

[27] Mahendradhata Y., Trisnantoro L., Listyadewi S., Soewondo P., Marthias T. (2017).

[28] Directorate General Of Disease Control And Environmental Sanitation (2016).

[29] UNICEF U Report Indonesia voice matters. https://indonesia.ureport.in/.

[30] UNICEF U Report Indonesia: engagement statistics. https://indonesia.ureport.in/engagement/.

[31] Claramita M., Fitriyani N., Syah N.A. (2021). Empowering adolescents as peer-educators for early prevention of non-communicable diseases: through existing 'POSBINDU' program in Indonesia. J Family Med Prime Care.

[32] Azzopardi P.S., Willenberg L., Wulan N. (2020). Direct assessment of mental health and metabolic syndrome amongst Indonesian adolescents: a study design for a mixed-methods study sampled from school and community settings. Glob Health Action.

[33] Braun V., Clarke V. (2006). Using thematic analysis in psychology. Qual Res Psychol.

[34] Republic of Indonesia Ministry of health (2020).

[35] Das S., Daxenberger L., Dieudonne L. (2020).

[36] Tuangratananon T., Wangmo S., Widanapathirana N. (2019). Implementation of national action plans on noncommunicable diseases, Bhutan, Cambodia, Indonesia, Philippines, Sri Lanka, Thailand and Viet Nam. Bull World Health Organ.

[37] Bonita R., Magnusson R., Bovet P. (2013). Country actions to meet UN commitments on non-communicable diseases: a stepwise approach. Lancet.

[38] United Nations Secretary-General (2015).

[39] Kraak V.I., Swinburn B., Lawrence M., Harrison P. (2014). An accountability framework to promote healthy food environments. Public Health Nutr.

[40] Widyaningsih V., Febrinasari R.P., Pamungkasari E.P. (2022). Missed opportunities in hypertension risk factors screening in Indonesia: a mixed-methods evaluation of integrated health post (POSBINDU) implementation. BMJ Open.

