# Supplementary material for: Towards responsive policy and actions to address non-communicable disease risks amongst adolescents in Indonesia: insights from key stakeholders
Source: Lancet Reg Health Southeast Asia. 2023 Aug 16;18:100260. doi: 10.1016/j.lansea.2023.100260 (PMC10667299; doi:10.1016/j.lansea.2023.100260)
Supplement: Appendix [file mmc1.docx]

# **Appendix.**

**Appendix 1. Bahasa Language Abstract.**

**Pendahuluan**

Penyakit tidak menular (PTM) seperti kanker, diabetes, penyakit jantung, gangguan jiwa dan penyakit paru kronis merupakan penyebab utama kematian dan kecacatan di Indonesia. Risiko untuk PTM muncul di usia remaja. Walau masa remaja merupakan waktu yang penting dalam tahap kehidupan untuk intervensi, kaum remaja sering kali dikesampingkan dalam kebijakan dan praktik mengenai PTM. Penelitian ini bertujuan untuk memahami bagaimana kebijakan dan praktik sebaiknya menanggulangi risiko PTM (merokok tembakau, aktivitas fisik yang rendah, dan diet) untuk remaja di Indonesia, dan bagaimana kalangan muda bisa ikut terlibat secara bermakna.

**Metode**

Penelitian ini menggunakan metode kualitatif wawancara mendalam melalui konferensi video (n=21) yang dilakukan dalam Bahasa Inggris atau Bahasa Indonesia, dengan pemangku kepentingan di Indonesia. Peserta meliputi pembuat kebijakan, mitra implementasi, dan advokat yang berfokus pada kesehatan remaja atau PTM. Wawancara direkam, ditranskrip, diterjemahkan, dan dianalisis secara tematis menggunakan NVivo12. Temuan penelitian disebarluaskan kepada peserta untuk validasi dan umpan balik. Peserta remaja (n=7) menghadiri lokakarya tambahan dan mempertimbangkan rekomendasi dan praktik yang dapat diambil dari penelitian ini.

**Hasil**

Para peserta menentukan bahwa organisasi pemerintah dan non-pemerintah telah menjalani praktik untuk menanggulangi PTM di Indonesia, tetapi hanya sedikit dari inisiatif yang ada menargetkan remaja, dan layanan untuk remaja jarang menangani risiko PTM. Para peserta juga merasa bahwa kebijakan untuk melindungi remaja dari risiko PTM (yaitu, area bebas asap rokok di tempat umum) tidak selalu ditegakkan. Untuk program atau inisiatif yang berfokus pada kesehatan remaja, program yang melibatkan remaja sebagai co-creator dan pemimpin dianggap lebih sukses. Dengan demikian, para peserta merekomendasikan keterlibatan kaum muda yang lebih bermakna, termasuk kesempatan bagi kaum muda untuk memimpin rangkaian inisiatif. Rekomendasi lainnya mencakup kebutuhan untuk keterlibatan lintas sektoral dan pendekatan ‘whole-of-government’ untuk pencegahan mengingat determinan faktor risiko PTM yang rumit, dan kebutuhan untuk praktik berbasis bukti yang didukung oleh data berkualitas untuk memungkinkan pemantauan kemajuan.

**Interpretasi**

Terdapat kebutuhan untuk memperkuat kebijakan dan praktik yang menanggulangi risiko PTM pada kalangan remaja di Indonesia. Penelitian ini menemukan bahwa keterlibatan bermakna yang memberi kesempatan pada kaum muda untuk memimpin, praktik lintas sektoral, dan pemberian tanggapan berdasarkan data yang berbasis bukti merupakan strategi-strategi utama.

**Pendanaan**

Kantor Regional UNICEF Asia Timur dan Pasifik.

## **Appendix 2. Full Interview Question Guide: English and Bahasa.**

Version 1 dated 16 November 2020.

| INTERVIEWER NAME: |  | DATE |  |
| --- | --- | --- | --- |
| START TIME: |  | END TIME: |  |
|  | | | |
| CITY/ PROVINCE: |  | SECTOR: |  |
| PARTICIPANT ROLE: |  | GENDER: |  |
| AGE GROUP: | 18-25 | 26-35 | 36+ |
| CONSENT FORM SIGNED? | | YES / NO | |

| Questions | |
| --- | --- |
| 1) Understanding of the nature and extent of NCD risks, amongst adolescents in Indonesia | What is your understanding of non-communicable diseases (NCDs) and are they important in Indonesia?  *Apa yang Anda ketahui/pahami mengenai penyakit tidak menular (PTM) dan apakah isu terkait PTM (yang disebutkan sebelumnya) penting di Indonesia?*  What do you know about the risk factors associated with NCDs?  *Apa yang Anda ketahui tentang faktor resiko yang terkait dengan PTM?*  (Prompt: There are a number of risk factors (such as smoking, obesity, diet, physical inactivity, and air pollution) that contribute to NCDs – what do you know about these risks?  *Ada beberapa faktor resiko (seperti merokok, diet tidak sehat, tidak aktif berolahraga, dan polusi udara) yang berkontribusi pada PTM – apa yang Anda ketahui tentang factor-faktor resiko ini?*  How important are these risks in adolescents (aged 10 to 19)?  *Seberapa penting resiko ini pada remaja usia 10-19 tahun?*  How do these NCD risks relate to the other issues of importance to adolescent health?  *Bagaimana faktor resiko ini terkait dengan masalah penting lainnya yang berhubungan dengan Kesehatan remaja?* |
| 2) Existing policies and programs of interest, including those with a youth-led approach. | What is being done currently to address NCD risk in Indonesia?  *Apa yang sedang dilakukan untuk mengatasi resiko PTM di Indonesia?*  (Prompt: you can speak about your field of interest/ at the local government level/ or nationally – whichever you are best placed to answer)  *Anda bisa berbicara tentang area yang Anda minati di tingkat pemerintah daerah atau secara nasional – apapun yang sifatnya paling relevan untuk Anda respon.*  Do any of these things directly target the adolescent population, ages 10-19? If not, could they be updated to include an adolescent-focus?  *Apakah di antara hal-hal yang Anda sebutkan secara langsung menargetkan remaja usia 10-19 tahun? Jika tidak, menurut Anda apakah hal-hal tersebut bisa direview sehingga focus pada remaja?*  Do you know of any **government policies** particularly focussed on NCD prevention?  *Apakah Anda mengetahui kebijakan pemerintah yang secara khusus fokus pada pencegahan PTM?*  (Prompt: For example, strategic plans and targets announced by government to reduce tobacco use; or a plan to introduce tobacco advertising laws)  *Misalnya, rencana strategis dan target yang diumumkan oleh pemerintah Indonesia untuk mengurangi penggunaan tembakau/merokok; atau rencana untuk memperkenalkan undang-undang periklanan tembakau.*  Were any of these **policies** adolescent focussed? Should they have an adolescent focus?  *Apakah ada dari kebijakan-kebijakan yang Anda sebutkan difokuskan pada remaja? Menurut Anda apakah kebijakan-kebijakan itu fokus pada remaja?*  Reflecting on these **government policies** on NCD prevention that you have mentioned - how effective are they? Can you think of any in particular that appear to be very effective/ ineffective for adolescents?  *Merefleksikan kebijakan pemerintah tentang pencegahan PTM yang Anda sebutkan – seberapa efektif kebijakan tersebut? Berikan contoh yang mungkin menurut Anda efektif atau tidak efektif untuk remaja?*  --- If relevant: Were there any **policies** which particularly focussed on Tobacco use, Overweight and obesity (healthy diet/ physical inactivity), Or Air pollution?  *Apakah ada kebijakan yang secara khusus fokus pada penggunaan tembakau, obesitas dan kegemukan (diet sehat/aktivitas fisik) atau polusi udara?*  Do you know of any current **initiatives or programs** being run which are particularly focussed on NCD prevention?  *Anda Anda mengetahui inisiatif atau program yang sedang dilakukan yang fokus pada pencegahan PTM?*  (Prompt: For example, an **initiative or program** run in workplaces or schools to encourage daily physical activity)  *Misalnya, inisiatif atau program yang dijalankan di tempat kerja atau sekolah untuk mendorong aktivitas fisik sehari-hari.*  Were any of these **initiatives** adolescent focussed? Could they have an adolescent focus in the future?  *Apakah program-program tersebut difokuskan pada remaja? Jika tidak, menurut Anda apakah program-program tersebut dapat berfokus pada remaja di masa mendatang?*  Were any of these **initiatives** led by a youth-run organisation or network?  *Apakah ada inisiatif atau program yang dipimpin oleh organisasi atau kelopok yang dijalankan oleh remaja?*  Reflecting on the NCD prevention **initiatives or programs** that you have mentioned - how effective are they? Can you think of any in particular that appear to be very effective/ ineffective for adolescents?  *Merefleksikan inisiatif atau program pencegahan PTM yang sudah Anda sebutkan – seberapa efektif kebijakan tersebut? Berikan contoh yang mungkin menurut Anda efektif atau tidak efektif untuk remaja?*  --- If relevant: What about the youth-led inititives specifically?  *Bagaimana dengan inisiatif atau program yang dijalankan oleh remaja secara khusus?*  --- If relevant: Were there any **initiatives or programs** which particularly focussed on Tobacco use, Overweight and obesity (healthy diet/ physical inactivity), Or Air pollution?  *Apakah ada inisiatif atau program yang secara khusus fokus pada penggunaan tembakau, obesitas dan kegemukan (diet sehat/aktivitas fisik) atau polusi udara?*  Thinking broader than NCD prevention now – do you know of any initiatives, programs, or campaigns to tackle an important health issue which have been largely youth-led or youth-focussed which have been particularly successful?  *Berfikir mengenai isu selain pencegahan PTM- apakah Anda mengetahui inisiatif, program, atau kampanye untuk menangani masalah penting kesehatan yang difokuskan pada remaja yang efektif dan menghasilkan dampak positif/sukses?*  (Prompt: for example you might know of a program run by young people which is focussed on reducing motorcycle injuries in adolescents by encouraging helmet use)  *Misalnya, Anda mungkin mengetahui program yang dijalankan oleh remaja yang berfokus untuk pengurangan cedera sepeda motor pada remaja dengan mendorong penggunaan helm.*  --- If relevant: What were the priority areas or goals for these initiatives? (e.g. HIV prevention, reducing pedestrian injury near schools, etc)  *Apa area prioritas atau tujuan dari inisiatif atau program tersebut? (Misalnya pencegahan HIV, mengurangi cedera pejalana kaki di dekat sekolah, dan lain-lain).* |
| 3) Strengths, gaps, opportunities, and challenges in NCD prevention policy/programming targeting adolescents. | What strengths and/or gaps do you see in current adolescent health and well-being initiatives, policies, or programs? (You can speak specifically about NCDs or other adolescent health topics)  *Apa strengths atau gaps yang Anda lihat dalam inisiatif, kebijakan, atau program kesehatan remaja saat ini? (Anda dapat berbicara tentang PTM secara khusus atau topik Kesehatan remaja lainnya)*.  Do you know of any future efforts (government, non-gov or collaborative) that are planned for promoting adolescent health and wellbeing? (Prompt: health promoting activities such as increasing exercise, reducing smoking)  *Apakaha Anda mengetahui usaha pemerintah atau NGOs atau kolaboratif antara pemerintah dan NGOs yang direncanakan untuk mempromosikan Kesehatan remaja? (Misalnya aktivitas promosi kesehatan seperti meningkatkan olahraga, mengurangi merokok)*.  What do you believe are the major challenges or barriers in developing policies or programs to promote adolescent health and well-being?  *Menurut Anda apa yang menjadi hambatan atau tantangan utama dalam mengembangkan kebijakan atau program promosi kesehatan pada remaja?*  Do you know of any programs which focus on training or capacity building of adolescents?  *Apakah Anda mengetahui program yang berfokus pada training/pelatihan atau peningkatan kapasitas remaja?*  --- If Yes: What did they receive training in? What kind of activities do they participate in?  *Di mana mereka menerima training? Jenis kegiatannya seperti apa?*  Are there any opportunities for UNICEF and young people to partner with you to improve NCD prevention policies and programs?  *Menurut Anda apakah ada kesempatan/peluang bagi UNICEF dan remaja untuk bermitra dengan Anda atau organisasi Anda untuk meningkatak kebijakan dan program pencegahan PTM?* |
| 4) Stakeholders, actors, and organisations that are (or could be in the future) active in NCD prevention among adolescents. | Are there any opportunities that you have had to engage with other partners (non-gov/ gov) on adolescent health or NCD prevention focussed initiatives?  *Apakah ada kesempatan di mana Anda terlibat dengan pemerintah atau NGOs untuk menjalankan inisiatif yang berfokus pada kesehatan remaja atau pencegahan PTM di masa lalu?*  --- If Yes: What were those opportunities?  *Beri contoh*.  Who are your key partners on adolescent health and NCD focussed initiatives? (Prompt: partners may include - Youth networks, UN agencies, NGO’s, [national or local] government departments/ sectors)  *Siapa mitra utama Anda dalam Kesehatan remaja dan inisatif berfokus pencegahan PTM? (Misalnya: UN agencies, organisasi remaja, NGOs, pemerintah daerah atau nasional)*.  ---For those who have partnered with youth networks: What was it like to partner with a youth-led organisation? (Prompt: Strengths/ weaknesses, what worked/ what didn’t)  *Bagi yang telah bermitra dengan organisasi remaja, bagaimana pendapat Anda bermitra dengan organisasi remaja? (Strengths/ weaknesses, what worked/ what didn’t)*.  Who do you feel should or could be further engaged? Be as specific as possible.  *Menurut Anda, siapa yang dapat diikutsertakan?*  Which platforms do you know of that are currently used to reach and engage with adolescents (and in what geographic areas)?  *Apakah Anda mengetahui platform apa yang saat ini digunakan untuk menjangkau dan melibatkan remaja?* |
| 5a) Perceptions and experiences of those in youth-led organisations  *If the interviewee is a young person in another org/ dept, skip 5a and do question set 5b* | How did you first get involved in the work that you are currently doing?  *Bagaimana Anda pertama kali terlibat dalam pekerjaan sekarang?*  Have you ever received any training, mentoring, or support that has helped you?  *Apakah Anda pernah menerima training, mentoring atau any kind of support yang telah membantu pekerjaan Anda?*  As a youth-led organisation, do you feel you have a ‘seat at the table’, are treated as an equal partner by other organisations/partners/gov?  *Sebagai organisasi remaja, apakah Anda merasa diperlakukan sebagai mitra yang setara oleh partners?*  Do you feel that you or your organisation can play a role in promoting healthier lifestyles/ reducing risk behaviour among young people in your community and country?  *Apakah Anda merasa bahwa Anda atau organisasi Anda dapat berperan dalam mempromosikan gaya hidup yang lebih sehat atau mengurangi perilaku beresiko di kalangan anak muda di komunitas atau negara Anda?*  Where do you feel you can have the biggest impact (family, school, community, peers, friends)?  *Menurut Anda, di mana Anda dapat memberikan pengaruh terbesar? (Keluarga, sekolah, komunitas, teman)*.  What support is your organisation/group receiving? (‘support’ e.g. technical support, donor support, etc…)?  *Dukungan apa yang diterima oleh organisasi Anda? (Technical support, finansial, atu lainnya)*.  What further supports could your organisation/group benefit from (e.g. technical support, donor support, etc…)?  *Dukungan lebih lanjut seperti apa yang menurut Anda dapat bermanfaat bagi organisasi Anda?*  What do you feel has been your organisations greatest achievement to date? (e.g. what have you done which has made the most impact?)  *Menurut Anda, apa pencapaian terbesar organisasi Anda hingga saat ini? (Misalnya apa yang telah Anda lakukan berdampak paling besar)*. |
| 5b) Perceptions and experiences of young people in other organisations | How did you first get involved in the work that you are currently doing? Have you ever received any training, mentoring, or support that has helped you?  *Bagaimana Anda pertama kali terlibat dalam pekerjaan sekarang? Apakah Anda pernah menerima training, mentoring atau any kind of support yang telah membantu pekerjaan Anda?*  As a young person, do you feel young people have a “seat at the table”, are treated as an equal partner by colleagues/ other organisations/ gov dept’s?  *Sebagai anak muda, apakah Anda merasa diperlakukan sama oleh pasangan atau rekan kerja Anda?*  Do you feel that young people can play a role in promoting healthier lifestyles/ reducing risk behaviour among young people in your community and country?  *Apakah Anda merasa bahwa kaum muda dapat berperan dalam mempromosikan gaya hidup yang lebih sehat atau mengurangi perilaku berisiko di antara kaum muda di komunitas atau negara Anda?*  Where do you feel young people can have the biggest impact (family, school, community, peers, friends)?  *Menurut Anda, di mana Anda dapat memberikan pengaruh terbesar? (Keluarga, sekolah, komunitas, teman)*.  What are some of the barriers to being a young person working in this field? What are some of the strengths?  *Menurut Anda, apa yang bisa menjadi kendala bagi kaum muda yang bekerja di bidang ini? Apa yang bisa menjadi plus/positif?* |
| 6) Youth Advisory Group | What do you think about a Youth-led approach to NCD prevention, specifically a Youth Stakeholder Group partnered with UNICEF and focussed on driving the NCD prevention agenda in Indonesia?  *Apa pendapat Anda tentang usaha atau pendekatan yang dipimpin oleh remaja untuk pencegahan PTM, khususnya a youth stakeholder group yang bermitra dengan UNICEF dan berfokus untuk mendorong agenda/usaha pencegahan PTM di Indonesia?*  Do you think this could be an effective strategy in Indonesia?  *Menurut Anda, apakah ini bisa menjadi strategi yang efektif di Indonesia?*  (Prompt: Could this work in Indonesia? Would relevant stakeholders engage with a Youth Led Group? What could be the Strengths or Barriers?)  *Apakah menurut Anda strategi ini bisa berhasil di Indonesia? Apakah stakeholders atau pemerintah Indonesia akan mengikutsertakan organisasi/group remaja? Kira-kira apa yang dapat menjadi nilai tambah/positif dan apa yang bisa menjadi hambatan?*  What could you see as potential priority actions for this group to work on initially?  *Menurut Anda apa aksi prioritas yang dapat dilakukan oleh organisasi/kelompok remaja pada awalnya?* |
| 7) Anything else? | Are there any other issues you would like to raise that we have not yet covered today?  *Apakah ada isu penting lain yang ingin Anda sampaikan yang mungkin belum kita bahas?*  I will go over a summary of what we have discussed, if you would like to add to or change anything you have said please let me know.  *Saya akan meringkas apa yang sudah kita diskusikan dan kalua Anda ingin menambah atau mengubah sesuatu yang sebelumnya Anda katakana tolong beri tahu saya.* |

## **Appendix 3. Participant approach letter and participant information and consent form.**

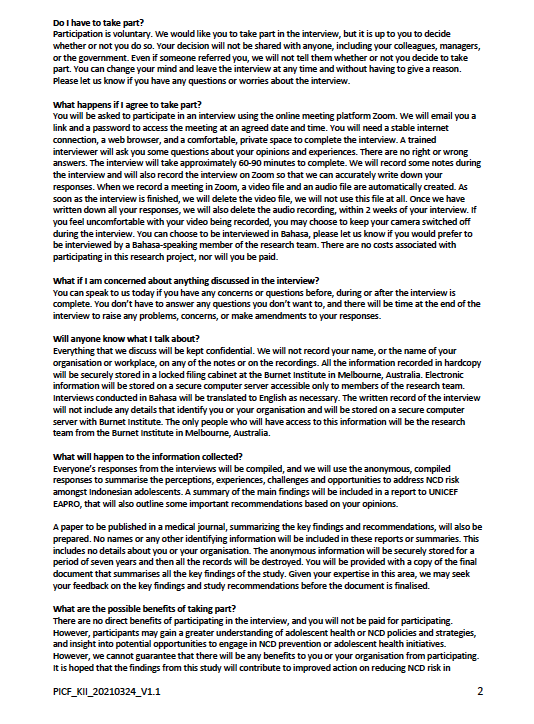


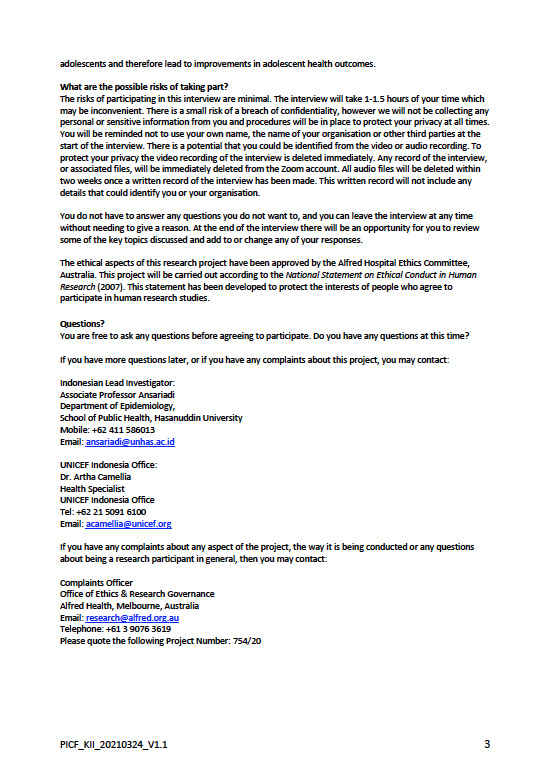


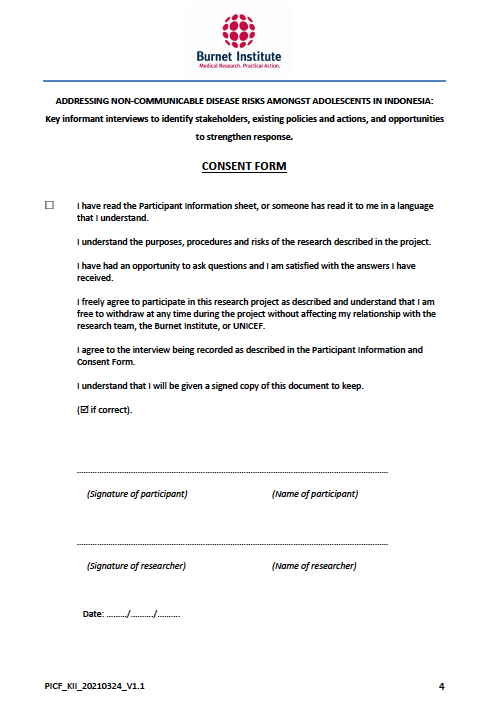


## **Appendix 4. Questions for workshop with youth participants.**

1) Is there anything missing in the findings?

Prompts: Do you feel like the findings are representative of your perceptions/ experience/ understanding of the situation in Indonesia? Have we represented your work adequately?

2) Are the draft recommendations complete?

Prompts: In your opinion do the draft recommendations address the findings? Is there anything else that you would add?

3) How do we practically action these?

Prompts: What are some concrete recommendations for moving forward!? What do you think of the ones that we have noted, as suggested in interviews?
